# A Designed Twist Sensor Based on the SPR Effect in the Thin-Gold-Film-Coated Helical Microstructured Optical Fibers

**DOI:** 10.3390/s22155668

**Published:** 2022-07-28

**Authors:** Mengwei Zhang, Lei Zhang, Qiang Chen, Ge Bai, Shuguang Li

**Affiliations:** 1 State Key Laboratory of Metastable Materials Science & Technology and Key Laboratory for Microstructural Material Physics of Hebei Province, School of Science, Yanshan University, Qinhuangdao 066004, China; zhangmw@stumail.ysu.edu.cn (M.Z.); cq2683648688@163.com (Q.C.); baige@stumail.ysu.edu.cn (G.B.); 2 School of Information Science and Engineering, Yanshan University, Qinhuangdao 066004, China; zhangl85@ysu.edu.cn

**Keywords:** helical microstructure optical fiber, surface plasmon resonance, twist sensing, high sensitivity

## Abstract

The traditional optical fiber-based twist sensing has the disadvantage of low sensitivity and difficulty of distinguishing the twist direction. Moreover, chiral isomerism may lead to sensing errors. In this paper, a six-hole helical microstructured optical fiber (HMSF) with a thin-gold-film-coat based on the surface plasmon resonance (SPR) effect was designed. The twist sensing characteristics of this fiber were further analyzed. Numerical calculation and analysis show that the combination of helical effect and SPR effect can design an HMSF-based sensor that is very sensitive to distortion. In the torsion range of ±300°, the distortion sensitivity can reach 2470.7 pm/(rad/m), and the linear correlation coefficient is 0.99996. Based on the special sensing mechanism, it has a good linear coefficient over a large range. Additionally, the direction of the twist can be easily discerned. The HMSF in this work not only has high sensitivity, high linearity, high fault tolerance rate, and a wide range of measurement, but is also easy to manufacture. Therefore, it is promising in the field of twist sensing and has a good application prospect.

## 1. Introduction

In recent years, with the rapid development of science and industry technology, twist sensing [1] and measurement of the distortion parameters [2] of various complex structures have become more and more important in industrial production, aerospace, safety monitoring, biomedicine, and other fields. Because of the big size, the application of traditional mechanical torque sensors is limited by difficult integration, easy corrosion, and other reasons [3]. At the same time, with the development of optical fiber sensing technology, optical fiber sensors have been widely studied in recent years due to their strong anti-electromagnetic interference ability, small size, easy embedding, corrosion resistance, and many other advantages [4]. In contortion sensing, traditional untwisted optical fiber-based sensors have low sensitivity, the twist direction is difficult to distinguish, and errors may also occur due to chiral isomerism. Helical microstructured fiber-based sensors are very sensitive, they can not only distinguish the twist direction intuitively but also have a large sensing range.

Surface plasmon resonance (SPR) exists on the interface between metal and dielectric, which is induced by charge density. In 1902, Wood et al. firstly discovered the SPR effect in an optical experiment, in which polarized light was irradiated on a metalized diffraction grating and an unusual reflection pattern was observed [5]. Optical fiber sensors based on the SPR effect have the advantages of high sensitivity, no trace, convenient to operate, and have been widely studied and applied in the fields of biosensing, environmental monitoring, food safety, and medical diagnosis [6,7,8,9]. In recent years, the SPR phenomenon has also been used in frontier fields such as biomedicine, cell research, and measurement of weak magnetic fields [10,11,12,13,14].

In 2010, Churikov et al. proposed and tested the chiral diffraction gratings in twisted fibers, proving that the resonance wavelength is not only caused by the diffraction of the core modes but also related to the twist rate and structural parameters [15]. In 2012, Russell et al. designed a novel waveguide, which was named helical microstructured fiber (HMSF) [16]. This work demonstrates the excitation of orbital angular momentum resonances in HMSF and provides new opportunities for optical control. Due to the helical symmetry structure, HMSF exhibits some unique properties. For example, the binding ability of the air hole to the fiber core will decrease with the increase in the helix, which makes the pitch *L* a bridge for sensing. In 2017, Wang Yiping et al. [17] prepared a helical long-period fiber grating through periodically torsional single-mode fiber. Compared with the long-period grating prepared by a traditional CO_2_ laser, this structure achieved an increase in distortion sensitivity of about 5 times, up to 1604 pm/(rad/m). In 2021, Li Jialong et al. [18] fabricated a six-hole large-aperture (air hole diameter > 10 μm) helical microstructured fiber. The HMSF in their paper has a simple structure and a good suppression of high-order modes.

Based on the transmission and SPR theory of optical fibers, a gold-plated six-hole HMSF was designed in this manuscript. The characteristics of the optical fiber are numerically simulated by using the COMSOL Multiphysics software with the finite element method. The aperture size and the thickness of the gold film are optimized for high-sensitivity twist sensing.

## 2. Fiber Structure and Calculation Methods

In this paper, a thin-gold-film-coated HMSF that can trigger the SPR effect is designed. Surface plasmon waves are generated by the interaction between the evanescent waves and the free electrons in the thin gold film [19,20]. When SPR is triggered, the intensity of the total reflection light is greatly reduced. The intuitive performance is that there will be an obvious absorption peak in the spectrum, and the corresponding wavelength is the resonance wavelength. At the same time, since the surface plasmon can only propagate near the metal film [21], the resonance wavelength is also very sensitive to the properties of the substance outside the gold film. We can also use the SPR effect to measure the refractive index of liquids [6,22,23,24]. Compared with the untwisted fiber, the higher-order modes can be attenuated more easily in the HMSF, and the transmitted light is mainly composed of a fundamental mode [22]. Moreover, the helical structure has a low binding ability to the core energy, which is conducive to the moderate spillover of the fundamental mode, thereby causing a strong SPR effect with the metal film.

### 2.1. Calculation Methods

The HMSF shows periodicity along the fiber axis, and the length corresponding to the period is called pitch *L*. As for the numerical simulation and analysis of helical fiber, the periodicity of the helical fiber can be used to simplify the calculation process. In 2008, A. Nicolet et al. removed one of the three coordinates by introducing a coordinate transformation [25], which achieved the substitution of 2D structures to periodic 3D structures and greatly reduced the amount of computation. In this paper, we also utilized similar mathematical methods to replace the 3D helical structure with a 2D anisotropic structure. The COMSOL Multiphysics software was used to simulate the helical fiber through the finite element method. A spiral coordinate system from the Cartesian coordinate system (*x*, *y*, *z*) was established to carry out coordinate transformation [25].

(1) $$\left\{\begin{array}{c} \xi_{1}=x\cos(\alpha z)-y\sin(\alpha z)\\ \xi_{2}=x\sin(\alpha z)+y\cos(\alpha z)\\ \xi_{3}=z \end{array}\right.$$

where *α* is the coefficient related to pitch *L*, and can be expressed as:

(2) $$\alpha =\frac{2\pi }{L}$$

The introduction of this helical coordinate system realizes that the periodicity coordinates of the helix can be expressed with the two basic vectors of *ξ*_1_ and *ξ*_2_. According to Formulas (1) and (3), the coordinate transformation is completed, and the matrix *T* for the transformation from the Cartesian coordinate system to the helical coordinate system can be obtained.

(3) $$J=\frac{\partial (\xi_{1},\xi_{2},\xi_{3})}{\partial (x,y,z)}$$

(4) $$T=\frac{J^{T}J}{\det(J)}=\left(\begin{array}{ccc} \alpha^{2}\xi_{2}{}^{2}+1 & -\alpha^{2}\xi_{1}\xi_{2} & -\alpha \xi_{2}\\ -\alpha^{2}\xi_{1}\xi_{2} & \alpha^{2}\xi_{1}{}^{2}+1 & \alpha \xi_{1}\\ -\alpha \xi_{2} & \alpha \xi_{1} & 1 \end{array}\right)$$

It is not difficult to find that the transformation matrix *T* is independent of *ξ*_3_. The fundamental vector *ξ*_3_ is eliminated in the matrix *T*, and in fact, the periodic characteristic of the optical fiber along the axis direction *z*(*ξ*_3_) was included in the constant *α* in this coordinate transformation operation. Thus, the 3D problem was simplified to a 2D problem. However, electromagnetic characteristics are not affected by the transformation of coordinates, and Maxwell’s equations are still valid. Therefore, the permittivity, permeability, and refractive index of helical fiber can be expressed as:

(5) ε′=εT

(6) μ′=μT

(7) $$n\prime =\sqrt{\varepsilon \prime \mu \prime }=nT$$

Based on the SPR effect, the resonance wavelength will shift regularly with the change in the pitch, so the variation in the pitch with the twist angle can be expressed as:

(8) $$L\prime (\theta )=\frac{360l}{360l+\theta L}\cdot L$$

where *l* represents the length of HMSF and *θ* represents the angle of twist.

The relationship between the twist angle and the resonance wavelength can be obtained by detecting the shift of the resonance wavelength. Like other types of sensors, wavelength sensitivity is an important parameter, expressed as [26]:

(9) $$S_{\lambda }=\frac{\Delta \lambda }{\Delta \theta }\mathrm{nm}\cdot {\mathrm{rad}}^{-1}\cdot m^{-1}$$

where ∆*λ* represents the displacement of resonance wavelength and ∆*θ* represents the change in torsion quantity.

### 2.2. Fiber Structure

**Figure 1:**
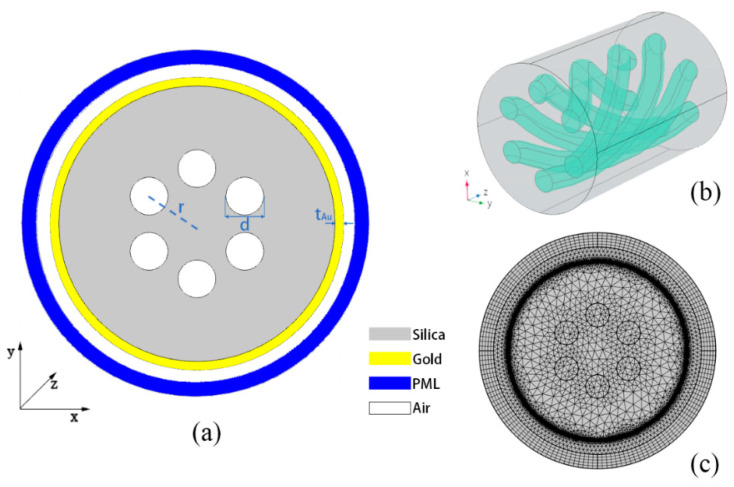
(**a**) Schematic diagram of the designed HMSF; (**b**) 3D structure diagram of the HMSF; (**c**) fiber diagram with a meshing grid.

The cross-section structure of the designed HMSF is shown in Figure 1a. The diameter of the HMSF is 125 μm, including six large-aperture air holes, the distance from the center of the air hole to the fiber center is *r* = 25 μm, and the diameter of the air holes is *d* = 17 μm. The outer surface of the fiber is coated with a thin gold film, which has a thickness of *t*_Au_ = 30 nm. The 3D helical structure of the optical fiber is shown in Figure 1b, and the pitch of the HMSF is set as *L* = 10 mm. Considering that the fiber is exposed to air during twist sensing and the influence of the material outside the gold film of the fiber cannot be ignored, a 10 μm air layer was added on the outside of the gold film in the simulation calculations with the COMSOL Multiphysics software. A PML layer was added outside the air layer as a boundary for the calculations. The meshing is shown in Figure 1c. The complete grid contains 213,530 domain units and 19,173 boundary elements. It should be noted that the initial value of the helix angle added for the fiber interior and the gold film is different in the calculation.

### 2.3. Experimental Device

**Figure 2:**
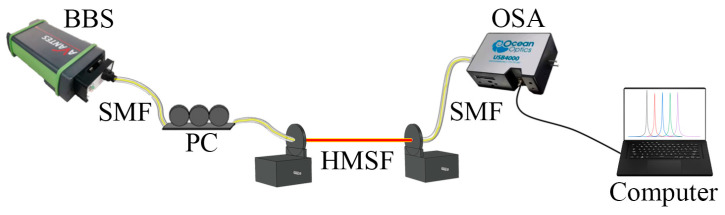
Schematic diagram of the experimental device.

The experimental device is shown in Figure 2 schematically. The whole system consists of a broadband light source (BBS), a polarization controller (PC), an optical spectrum analyzer (OSA), two rotatable optical fiber clamps and a computer. During the experiment, the HMSF should be welded with the jumpers at both ends, then fixed on the fixture. An HMSF that is slightly longer than the required length is needed to ensure the distance between the two fixtures is correct. Special care should be taken to make sure that the fixtures are firmly fixed to HMSF, thereby all the twist force applied later by the fixtures can be put on the HMSF as much as possible. By satisfying these details in experiment, the potential interference resulting from the twisted jumper without a helix at both ends can be eliminated. In addition, the torsional strength of the fiber fusion point does not become a bottleneck of the measurement. After the light emitted by the BBS passes through the polarization controller, only one suitable polarization direction is retained to make the light propagate into the helical microstructure fiber (HMSF). The emergent light is received by the OSA at the other end and finally detected by a computer. The helix to the fiber can be applied by slowly adjusting the knob on the fixtures. We can observe and record the spectral changes under different twist angles, and then process the data to obtain the twist sensitivity of the sensor.

## 3. Results and Discussion

### 3.1. Structure Optimization

**Figure 3:**
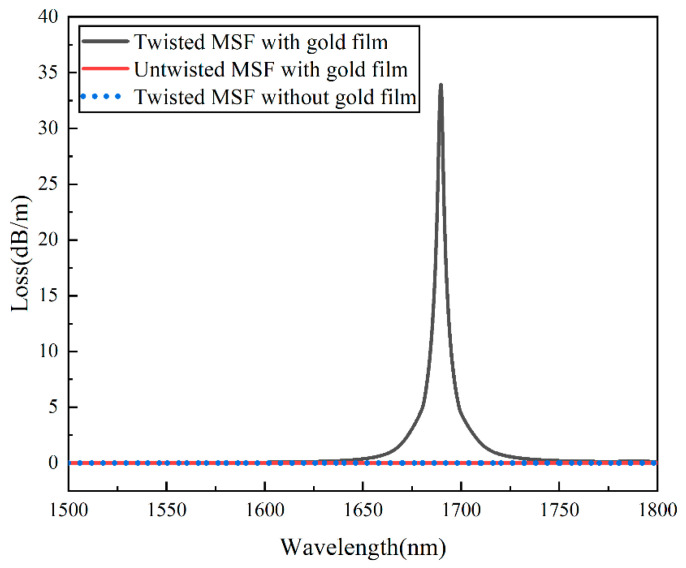
Comparison of the losses of HMSF with gold film, untwisted MSF with gold film, and HMSF without gold film.

According to the initial structural parameters given in Figure 1, the confinement losses for the gold-film-coated HMSF, untwisted MSF with gold film, and HMSF without gold film are calculated, respectively. The comparison of the losses is shown in Figure 3. The untwisted MSF has a strong binding ability so the energy in the fiber core cannot leak to the cladding area and cannot interact with the gold film on the outer surface to trigger the SPR effect. The confinement loss curve for the untwisted MSF with gold film exhibits a flat low loss on the transmission line without loss peaks. The binding ability is weakened by the helical structure, and more energy can leak from the core to the cladding region. However, the twisted MSF without gold film lacks the necessary conditions to stimulate the SPR effect, and the confinement loss curve has no loss peak. For the gold-film-coated HMSF, the SPR effect can be stimulated by the interaction between the leaked energy and the gold film. Thereby, an obvious peak appears in the confinement loss curve. The SPR effect in the gold-film-coated six-hole HMSF is further simulated to investigate the twist sensing properties.

**Figure 4:**
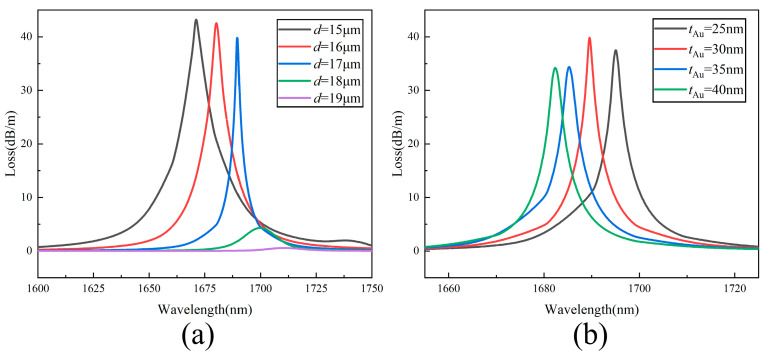
(**a**) Confinement losses for different air hole sizes *d*; (**b**) confinement loss for different thicknesses of the gold film.

In order to analyze the influence of the structural parameters on the confinement loss of the gold-film-coated HMSF, the pore diameters *d* or the gold film thicknesses *t*_Au_ are adjusted while other parameters remain the same. The confinement losses for the gold-film-coated HMSF with the same gold film thicknesses *t*_Au_ = 30 nm but different air hole diameters *d* are shown in Figure 4a. When *d* ≥ 18 μm, the air hole has a strong energy binding ability, which restricts the escape of light from the core to the cladding, resulting in a weak SPR effect and inconspicuous loss peaks. When 15 μm ≤ *d* ≤ 17 μm, the narrowing of the air hole weakens the cladding’s restraint, thereby allowing more energy to escape from the core to the cladding region. The light in the cladding is totally reflected to the surface of the outer gold film and interacts with the free electrons, then the surface plasmon polaritons (SPP) mode is generated. When the refractive indices of the fundamental mode and the SPP mode reach the matching point, the excitation of the surface plasmon mode on the surface of the gold film produces an obvious loss peak. With the increase in the air hole diameter, the effective refractive index of the core decreases, the phase changes, and the position of the loss peak will red-shift. When *d* increases from 15 to 17 μm, the confinement of the air hole to the core increases, and the difficulty of phase matching increases, eventually resulting in a decrease in the full width at half maximum of the loss peak.

The confinement losses for the gold-film-coated HMSF with the same air hole diameters *d* = 17 μm but different gold film thicknesses *t*_Au_ are shown in Figure 4b. The position of the loss peak blueshifts with the increase in gold film thickness. The full widths at half maximum of the loss peaks are very close with the thickness of the gold film settled in the range of 25–40 nm. For the case of *t*_Au_ = 30 nm, the loss curve has the highest peak, which is beneficial for sensing detection. Therefore, the 30 nm gold film thickness was selected for further study.

**Figure 5:**
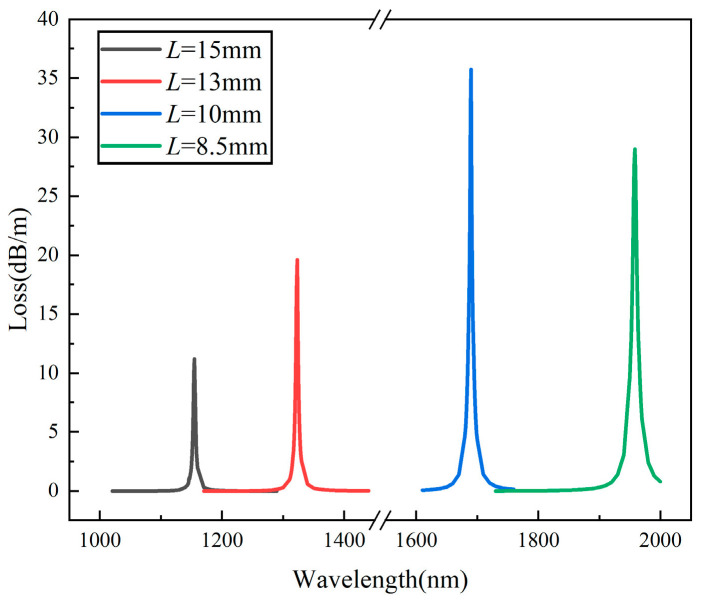
Confinement losses for different pitch *L*.

In addition, it is important to choose a suitable initial pitch *L*, since the characteristics of HMSF are highly related to the pitch. The confinement losses for the gold-film-coated HMSF with the same air hole diameters *d* = 17 μm, the same gold film thicknesses *t*_Au_ = 30 nm, but different pitch *L* are shown in Figure 5. When the pitch L is large, that is, the helix is small, the air hole still has a strong binding ability to the fiber core, and only a small amount of energy can overflow to trigger the SPR effect with the gold film, so the loss peak is low. When the helical pitch *L* is small, although a good loss peak can still be generated, the resonance wavelength in the sensing will exceed 2000 nm, which is beyond the range of general light sources and is not conducive to experimental research. Therefore, the pitch *L* = 10 mm was selected for the following research.

### 3.2. Coupling Pattern Analysis

**Figure 6:**
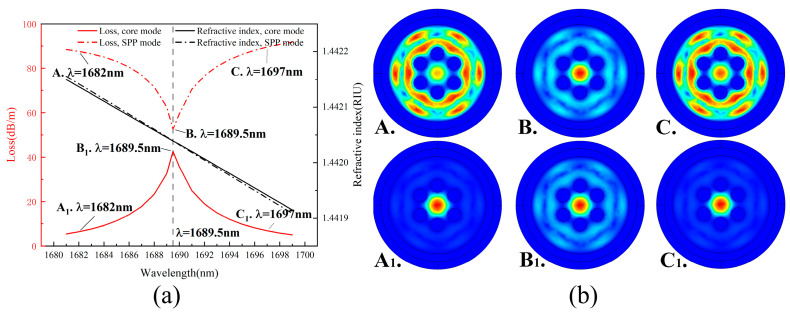
(**a**) Coupling relation diagram between the SPP mode and the fundamental mode; (**b**) schematic diagram of the mode field of SPP and fundamental modes in the coupled band.

When the gold film thickness is 30 nm and the air hole diameter is 17 μm, the confinement loss diagram of the designed HMSF is shown in Figure 6a. At the effective refractive index intersection of the SPP mode and the core mode, the core mode couples with the SPP mode, resulting in a larger loss peak in the confinement loss of the core mode. The core mode electric field distribution diagrams (A_1_–C_1_) and the corresponding SPP mode electric field distribution diagrams (A–C) at wavelengths of 1682, 1689.5, and 1697 nm are shown in Figure 6b, and the corresponding wavelength points are marked in Figure 6a.

### 3.3. Sensing Performance Analysis

**Figure 7:**
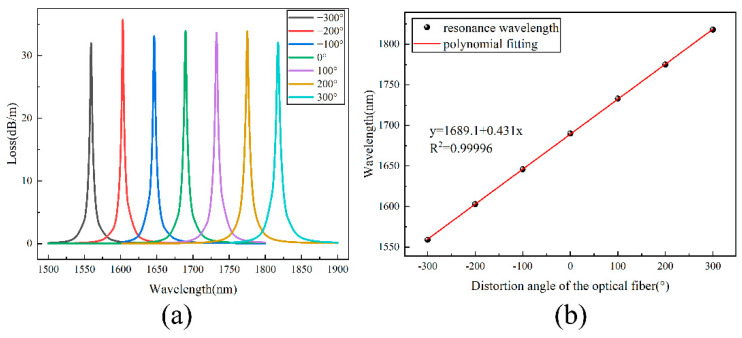
(**a**) Loss spectra of HMSF with different additional torpor; (**b**) fitting relation between the resonance wavelength and the additional torsion angle.

The loss spectra of the fundamental modes of the gold-film-coated HMSF with the additional twisting changing from −300° to +300° are shown in Figure 7a. The wavelength of the loss peak shifts with the change in the twisting angle and direction. With the increase in the twist angle along the positive direction, the resonance wavelength is red-shifted. When the twist angle increases along the negative direction, the resonance wavelength is blue-shifted. Taking the initial state as the reference value (0°), the linear fitting result of the sensitivity is shown in Figure 7b. It presents the relationship between the twist angle and the wavelength of the loss peak, and the linear fitting formula is as follows:

(10) y=0.431x+1689.1

The sensitivity of the gold-film-coated HMSF is 431 pm/° and the linear correlation coefficient is 0.99996. Since the selected fiber length is 10 cm, the sensitivity can be converted to 43.1 pm/(°/cm), that is, 2470.7 pm/(rad/m). To sum up, the twist sensor can not only identify the twist direction but also has a good linearity.

### 3.4. Comparison and Analysis

The performances of the HMSF sensor proposed in this paper are compared with other existing fiber optic sensors for twist sensing, as shown in Table 1. It can be found that the sensitivity and linearity of this sensor are both greatly improved. Therefore, the gold-film-coated HMSF proposed in this paper provides a new direction for further research and has good potential in the field of fiber distortion sensors.

## 4. Conclusions

In this paper, a gold-film-coated HMSF-based sensor was designed, which achieved twist sensing with extremely high sensitivity by exploiting the loss peak excited by the SPR effect. It has the advantages of good linearity and distinguishable torsion direction compared with the other sensors. Sensitivity to pitch changes is one of the characteristics of the HMSF, and it is feasible to coat the outer surface to produce a distinct loss peak by the SPR effect. In the twisting changing from −300° to +300°, the sensitivity can reach 2470.7 pm/(rad/m), and the linear correlation coefficient is 0.99996. The wavelength sensitivity can maintain good linearity in a wide range. The structure of the HMSF is simple, the preparation is easy, and this HMSF has certain fault tolerance. Therefore, it has application potential in the fields of safety detection and industrial production.

## Figures and Tables

**Table 1:** Comparison of distortion sensing results with other sensing results.

Structural Features	Wavelength Sensitivity (pm/(rad/m))	Features
Ultra-Long Period Fiber Grating [26]	224.4	Good linearity, can distinguish twist direction
PM Sagnac Ring [27]	Max 1464.7	Non-linear, cannot distinguish twist direction
Mach–Zendel interferometer based on pre-twisted fiber [28]	Max 1035	Non-linear, can distinguish the torsion direction, and the sensitivity in the positive and negative directions is different
Long Period Fiber Grating [17]	1604	Good linearity, can distinguish the twist direction
HMSF with outer gold coating (this paper)	2470.7	Good linearity, can distinguish twist direction

## Data Availability

The data presented in this study are openly available.
